# Supplementary material for: A novel method for estimating properties of attentional oscillators reveals an age-related decline in flexibility
Source: eLife. 2024 Jun 21;12:RP90735. doi: 10.7554/eLife.90735 (PMC11192533; doi:10.7554/eLife.90735)
Supplement: Supplementary file 1. — (a) Descriptive statistics and test results for comparison of β estimates against null distributions in Experiment 1 analyses. (b) Descriptive statistics of unpaced tapping measures in first and second experiments, and test results for pairwise comparisons. (c) Results of the pairwise correlation analyses between preferred rate estimates from each task and condition in Experiment 2. (d) Descriptive statistics and test results for comparison of β estimates against null distributions in Experiment 2 analyses. [file elife-90735-supp1.docx]

**Supplementary file 1a**

| **Session type** | **Predictor** | **Outcome** | **M** | **SD** | **T** | **df** | **p** |
| --- | --- | --- | --- | --- | --- | --- | --- |
| **Linear-order session** | \|-DEV\| | accuracy | 1.397 | 0.588 | 12.115 | 25 | 2.9269e-12 |
|  | \|+DEV\| | accuracy | 1.118 | 0.430 | 13.253 | 25 | 4.1489e-13 |
| **Random-order session** | \|-DEV\| | accuracy | 0.672 | 0.315 | 11.083 | 26 | 1.1939e-11 |
|  | \|+DEV\| | accuracy | 0.514 | 0.271 | 9.668 | 25 | 3.1518e-10 |
|  | \|-𝚫IOI\| | accuracy | -0.165 | 0.138 | -6.096 | 25 | 1.1323e-06 |
|  | \|+𝚫IOI\| | accuracy | -0.138 | 0.126 | -5.603 | 25 | 3.9471e-06 |

**Supplementary file 1b**

| **Experiment 1** | | | | | | | | | | | | |
| --- | --- | --- | --- | --- | --- | --- | --- | --- | --- | --- | --- | --- |
| **Session averages** | **Random-order** | | **Linear-order** | |  | **paired-samples**  **t-test** | | |  | **Pearson correlation** | | |
|  | **M** | **SD** | **M** | **SD** |  | **T** | **df** | **p** |  | **r** | **df** | **p** |
| **SMT (s.)** | 0.555 | 0.189 | 0.189 | 0.194 |  | -1.763 | 26 | .090 |  | .865 | 25 | 5.8512e-09 |
| **Measurement** | **1 (before)** | | **2 (after)** | |  |  |  |  |  |  |  |  |
|  | **M** | **SD** | **M** | **SD** |  |  |  |  |  |  |  |  |
| **Random-order session** | | | | | | | | | | | | |
| **SMT (s.)** | 0.534 | 0.199 | 0.563 | 0.214 |  | -0.4174 | 25 | .67991 |  | .549 | 24 | 0.0037158 |
| **FMT ‘slowest’ (s.)** | 1.442 | 0.465 | 1.461 | 0.522 |  | 0.0202 | 24 | .98403 |  | .493 | 23 | 0.012206 |
| **FMT ‘fastest’ (s.)** | 0.227 | 0.059 | 0.223 | 0.045 |  | -0.0396 | 25 | .96873 |  | .737 | 24 | 1.747e-05 |
| **Linear-order session** | | | | | | | | | | | | |
| **SMT (s.)** | 0.574 | 0.181 | 0.605 | 0.249 |  | -0.8031 | 26 | .42919 |  | .613 | 25 | 0.00068186 |
| **FMT ‘slowest’ (s.)** | 1.602 | 0.575 | 1.628 | 0.639 |  | -0.5583 | 24 | .58179 |  | .627 | 23 | 0.00080427 |
| **FMT ‘fastest’ (s.)** | 0.211 | 0.051 | 0.204 | 0.037 |  | 0.2351 | 23 | .81621 |  | .611 | 22 | 0.001531 |
| **Experiment 2** | | | | | | | | | | | | |
| **SMT (s.)** | 0.598 | 0.222 | 0.669 | 0.218 |  | -2.6163 | 31 | .013613 |  | .758 | 30 | 5.1511e-07 |
| **FMT ‘slowest’ (s.)** | 1.427 | 0.634 | 1.438 | 0.713 |  | -0.1195 | 28 | .90577 |  | .704 | 27 | 2.8679e-05 |
| **FMT ‘fastest’ (s.)** | 0.256 | 0.078 | 0.261 | 0.067 |  | -0.5938 | 28 | .55742 |  | .669 | 27 | 7.2455e-05 |

**Supplementary file 1c**

|  | | **SMT #1** | **SMT #2** | **Duration**  **discrimination** | **Paced**  **tapping** | **Slider #1**  **fast-start** | **Slider #1**  **slow-start** | **Slider #2**  **fast-start** | **Slider #2**  **slow-start** | **Keypress**  **fast-start** |
| --- | --- | --- | --- | --- | --- | --- | --- | --- | --- | --- |
| **Keypress**  **slow-start** | r = | 0.19238738 | 0.14704527 | -0.2777905 | 0.2208119 | 0.35880437 | **0.51559653** | **0.46750895** | **0.50924355** | 0.04852623 |
|  | p = | 0.29979413 | 0.42989342 | 0.15235564 | 0.24969911 | 0.07817678 | **0.00498272** | **0.0105523** | **0.00564496** | 0.80260746 |
|  | df = | 29 | 29 | 26 | 27 | 23 | **26** | **27** | **26** | 27 |
| **Keypress**  **fast-start** | r = | -0.1991552 | 0.05101907 | -0.2435522 | 0.08757407 | **0.49992895** | 0.35375669 | 0.26744148 | 0.09020309 |  |
|  | p = | 0.29138758 | 0.78889695 | 0.22088111 | 0.65767596 | **0.01286023** | 0.07025848 | 0.16886475 | 0.64804425 |  |
|  | df = | 28 | 28 | 25 | 26 | **22** | 25 | 26 | 26 |  |
| **Slider #2**  **slow-start** | r = | 0.16208113 | 0.3406378 | -0.0602415 | -0.0044475 | 0.3208116 | **0.4810407** | **0.52137264** |  |  |
|  | p = | 0.40089994 | 0.07057311 | 0.77003349 | 0.98243502 | 0.12639402 | **0.01108119** | **0.00443913** |  |  |
|  | df = | 27 | 27 | 24 | 25 | 22 | **25** | **26** |  |  |
| **Slider #2**  **fast-start** | r = | 0.35569764 | **0.52790234** | -0.0334548 | 0.17471004 | **0.79673077** | **0.46904928** |  |  |  |
|  | p = | 0.05372163 | **0.00271543** | 0.86842739 | 0.37389759 | **1.1156e-06** | **0.01026563** |  |  |  |
|  | df = | 28 | **28** | 25 | 26 | **24** | **27** |  |  |  |
| **Slider #1**  **slow-start** | r = | 0.28046384 | **0.36893725** | -0.3237635 | 0.19619323 | **0.69076946** |  |  |  |  |
|  | p = | 0.14057008 | **0.04889556** | 0.1066389 | 0.32669799 | **9.3667e-05** |  |  |  |  |
|  | df = | 27 | **27** | 24 | 25 | **24** |  |  |  |  |
| **Slider #1**  **fast-start** | r = | 0.13779212 | 0.32128253 | -0.3521865 | 0.16391112 |  |  |  |  |  |
|  | p = | 0.50205298 | 0.10950586 | 0.09932549 | 0.42364447 |  |  |  |  |  |
|  | df = | 24 | 24 | 21 | 24 |  |  |  |  |  |
| **Paced tapping** | r = | **0.41214417** | **0.40505774** | -0.0166448 |  |  |  |  |  |  |
|  | p = | **0.02362701** | **0.02638676** | 0.93432732 |  |  |  |  |  |  |
|  | df = | **28** | **28** | 25 |  |  |  |  |  |  |
| **Duration discrimination** | r = | **0.48378586** | **0.4353227** |  |  |  |  |  |  |  |
|  | p = | **0.00783814** | **0.0182627** |  |  |  |  |  |  |  |
|  | df = | **27** | **27** |  |  |  |  |  |  |  |
| **SMT #2** | r = | **0.75762652** |  |  |  |  |  |  |  |  |
|  | p = | **5.1511e-07** |  |  |  |  |  |  |  |  |
|  | df = | **30** |  |  |  |  |  |  |  |  |

**Supplementary file 1d**

| **Task** | **Predictor** | **Outcome** | **M** | **SD** | **T** | **df** | **p** |
| --- | --- | --- | --- | --- | --- | --- | --- |
| **Duration**  **discrimination** | \|-𝚫IOI\| | accuracy | -0.185 | 0.279 | -3.5685 | 28 | 0.0006593 |
|  | \|+𝚫IOI\| | accuracy | -0.230 | 0.261 | -4.7417 | 28 | 2.8093e-05 |
| **Paced tapping** | \|-𝚫IOI\| | \|TME\| | 0.004 | 0.005 | 4.7222 | 25 | 3.8278e-05 |
|  | \|+𝚫IOI\| | \|TME\| | 0.004 | 0.008 | 2.493 | 29 | 0.0093111 |
