# Supplementary material for: A novel method for estimating properties of attentional oscillators reveals an age-related decline in flexibility
Source: eLife. 2024 Jun 21;12:RP90735. doi: 10.7554/eLife.90735 (PMC11192533; doi:10.7554/eLife.90735)
Supplement: Supplementary file 2. [file elife-90735-supp2.docx]

| **Welcome!** | **Willkommen** |
| --- | --- |
| In this experiment, you will be asked to do several tasks: | In diesem Experiment werden Sie gebeten, mehrere Aufgaben zu erledigen: |
| tapping your finger on the desk without hearing any sounds. | tippen mit dem Finger auf den Tisch, ohne ein Geräusch zu hören. |
| tapping your finger on the desk along with rhythms that you will listen to over headphones. | tippen mit dem Finger auf den Tisch zu den Rhythmen, die Sie über die Kopfhörer hören. |
| listening to rhythms and comparing time intervals. | Rhythmen hören und Zeitabstände vergleichen Hören von Rhythmen und Vergleichen von Zeitabständen. |
| adjusting the tempo of a rhythm. | Anpassen des Tempos eines Rhythmus. |
| Press ENTER to proceed. | Drücken Sie die EINGABETASTE, um fortzufahren. |

| **FREE TAPPING** | **TIPPEN OHNE GERÄUSCH** |
| --- | --- |
| In this section, you will be asked to tap your finger on the desk without hearing any sounds. | In diesem Abschnitt werden Sie gebeten, mit dem Finger auf den Tisch zu tippen, ohne ein Geräusch zu hören. |
| First, you will tap at a comfortable rate, then at the slowest rate, finally at the fastest rate that is comfortable for you to maintain. | Tippen Sie zunächst mit einer angenehmen Geschwindigkeit, dann mit der langsamsten und anschließend mit der schnellsten Geschwindigkeit, die Sie bequem beibehalten können. |
| Please press the right arrow key to go to next page. | Bitte drücken Sie die rechte Pfeiltaste, um zur nächsten Seite zu gelangen. |
| The expected duration for TIPPEN OHNE GERÄUSCH task is ~3 minutes | Die voraussichtliche Dauer der Aufgabe TIPPEN OHNE GERÄUSCH beträgt ~3 Minuten |
| Throughout the section, | Im gesamten Abschnitt |
| Please tap next to the microphone, within the area marked with tape. | Bitte tippen Sie neben dem Mikrofon in dem mit Klebeband markierten Bereich. |
| Please tap with only one finger. Choose the finger that is most comfortable for you to tap with. | Bitte tippen Sie nur mit einem Finger. Wählen Sie den Finger, mit dem Sie am bequemsten tippen können. |
| Please tap loud enough so that the microphone can pick up the taps. | Bitte tippen Sie so laut, dass das Mikrofon das Tippen wahrnehmen kann. |
| Please tap with equal strength. | Bitte tippen Sie mit gleicher Stärke. |
| Please make sure your clothes or accessories do not make sounds. | Bitte achten Sie darauf, dass Ihre Kleidung oder Accessoires keine Geräusche verursachen. |
| Please tap as evenly as possible. | Bitte tippen Sie so gleichmäßig wie möglich. |
| If you have questions, please notify the experimenter. | Wenn Sie Fragen haben, wenden Sie sich bitte an den Versuchsleiter. |
| Otherwise, please press ENTER to proceed to the task. | Andernfalls drücken Sie bitte die EINGABETASTE, um mit der Aufgabe fortzufahren. |
| When you see the grey screen, please tap your finger on the desk at a rate that is comfortable for you to maintain. | Wenn Sie den grauen Bildschirm sehen, versuchen Sie bitte mit dem Finger auf den Tisch zu tippen mit einer Geschwindigkeit, die für Sie angenehm ist. |
| You can stop tapping when the screen changes color. | Sie können mit dem Tippen aufhören, wenn sich die Farbe des Bildschirms ändert. |
| Press ENTER when you are ready. | Drücken Sie die EINGABETASTE, wenn sie bereit sind. |
| When you see the grey screen, please tap your finger at the slowest rate that is comfortable for you. | Wenn Sie den grauen Bildschirm sehen, tippen Sie bitte mit der LANGSAMSTEN Geschwindigkeit, die für Sie am angenehmsten ist. |
| When you see the grey screen, please tap your finger at the fastest rate that is comfortable for you. | Wenn Sie den grauen Bildschirm sehen, tippen Sie bitte mit der SCHNELLSTEN Geschwindigkeit, die für Sie am angenehmsten ist. |

| **SOUND VOLUME** | **EINSTELLUNG DER LAUTSTÄRKE** |
| --- | --- |
| Now, please wear the headphones and adjust them for your comfort. | Setzen Sie bitte jetzt die Kopfhörer auf und stellen Sie diese auf Ihren Komfort ein. |
| In this section, you will adjust the sound volume. Note that this level cannot be changed later. | In diesem Abschnitt stellen Sie die Lautstärke des Tons ein. Beachten Sie, dass dieser Wert später nicht mehr geändert werden kann. |
| Click on different locations on the slider to change the sound volume. You will hear a sound every time you change the volume. | Klicken Sie auf verschiedene Stellen des Schiebereglers, um die Lautstärke zu ändern. Sie hören jedes Mal einen Ton, wenn Sie die Lautstärke ändern. |
| Please press ENTER to start adjusting the volume. | Bitte drücken Sie die EINGABETASTE, um mit der Einstellung der Lautstärke zu beginnen. |
| Click on the left button  to CHANGE the sound volume | Klicken Sie auf die linke Taste  um die Lautstärke zu VERÄNDERN |
| Click on the right button  to SET the sound volume | Klicken Sie auf die rechte Taste  um die Lautstärke EINZUSTELLEN |
| Please DO NOT click and drag the mouse at the same time. Move the mouse and then click on different locations on the slider. | Bitte klicken und ziehen Sie die Maus NICHT gleichzeitig. Bewegen Sie die Maus und klicken Sie dann auf verschiedene Stellen des Schiebereglers. |

| **TAPPING TO RHYTHM** | **TIPPEN ZUM RHYTHMUS** |
| --- | --- |
| In the current section, you will be instructed about the task.  Please read the instructions carefully. | Im aktuellen Abschnitt werden Sie in die Aufgabe eingewiesen.  Bitte lesen Sie die Anweisungen sorgfältig durch. |
| You can travel between pages with the arrow keys freely until the practice section. | Bis zum Übungsteil können Sie mit den Pfeiltasten frei zwischen den Seiten wechseln. |
| The expected duration for TAPPING TO RHYTHM task is ~ 15 minutes (7,5 minutes x 2 blocks with a break in between) | Die voraussichtliche Dauer für die Aufgabe TIPPEN ZUM RHYTHMUS beträgt ~ 15 Minuten (7,5 Minuten x 2 Blöcke mit einer Pause dazwischen) |
| On each trial, you will hear a sequence of five sounds. | Bei jedem Durchgang hören Sie eine Folge von fünf Töne. |
| Your task is to start tapping along with the sounds, and then keep going at the same rate once the sounds stop. | Ihre Aufgabe ist es, mit den Tönen zu tippen und dann mit der gleichen Geschwindigkeit weiterzumachen, sobald die Töne aufhören. |
| You should keep tapping as evenly as possible until the screen changes color. | Sie sollten so gleichmäßig wie möglich tippen, bis der Bildschirm seine Farbe ändert. |
| Here is a graphical representation of the task. Remember, you should start tapping your finger soon as you can and tap along with the sounds. Keep tapping after the sounds stop. | Hier sehen Sie eine graphische Darstellung der Aufgabe. Denken Sie daran, dass Sie so schnell wie möglich anfangen mit dem Finger zu tippen, sobald Sie mit den Tönen mittippen können. Tippen Sie weiter, nachdem die Töne aufgehört haben. |
| You have completed the instructions. If you have understood the task, please press ENTER to proceed to the practice section. | Sie haben die Anweisungen abgeschlossen. Wenn Sie die Aufgabe verstanden haben,  drücken Sie bitte EINGABETASTE, um mit dem Übungsteil fortzufahren. |
| **PRACTICE** | **ÜBUNG** |
| In this section, you will practice the task for at least 6 trials. After each trial, you will be given feedback regarding your performance. | In diesem Abschnitt üben Sie die Aufgabe für mindestens 6 Durchgänge. Nach jedem Versuch erhalten Sie eine Rückmeldung zu Ihrer Leistung. |
| Press ENTER to start the practice trials. | Drücken Sie die EINGABETASTE, um die Übungsversuche zu starten. |
| **FEEDBACK** | **RÜCKMELDUNG** |
| Something went wrong. Please notify the experimenter! | Etwas ist schiefgelaufen. Bitte benachrichtigen Sie die Versuchsleiterin! |
| You did not start tapping before the sounds ended. Please try to start tapping as soon as you hear the sounds. | Sie schienen nicht zu tippen, bevor der Ton aufgehört hat. Bitte versuchen Sie zu tippen, sobald Sie den Ton hören. |
| Press ENTER to proceed. | Drücken Sie die EINGABETASTE, um fortzufahren. |
| Not enough taps were recorded. Please do not stop tapping until the screen changes color. | Es wurden nicht genügend Tippgeräusche aufgezeichnet. Bitte hören Sie nicht auf zu tippen bis der Bildschirm seine Farbe ändert. |
| You were too fast! | Sie waren zu schnell! |
| You were too slow! | Sie waren zu langsam! |
| Let’s try another. | Versuchen wir es mit einem anderen. |
| Good job! | Gut gemacht! |
| You have completed the practice section.  If you have questions, please notify the experimenter. | Sie haben den Übungsteil abgeschlossen. Wenn Sie noch Fragen haben, informieren Sie bitte die Versuchsleiterin. |
| Otherwise, please press ENTER to proceed to the main task. | Andernfalls drücken Sie bitte die EINGABETASTE, um mit der Hauptaufgabe fortzufahren. |
| Note that no feedback will be given throughout this section. | Beachten Sie, dass es keine Rückmeldung während des Experiments geben wird. |
| You have successfully completed the TAPPING TO RHYTHM section. | Sie haben erfolgreich den Abschnitt tippen ZUM RHYTHMUS abgeschlossen. |
| You can take a short break before the next section. | Vor dem nächsten Abschnitt können Sie eine kurze Pause einlegen. |
| Press ENTER to end the break  and proceed to the next section. | Drücken Sie die EINGABETASTE, um die Pause zu beenden  und fahren Sie mit dem nächsten Abschnitt fort. |

| **DURATION DISCRIMINATION** | **DAUERDISKRIMINIERUNG** |
| --- | --- |
| In the current section, you will be instructed about the task. Then, you will hear some examples. Finally, before the main task, you will practice the task. Please read the instructions carefully. | Im aktuellen Abschnitt werden Sie in die Aufgabe eingewiesen. Dann werden Sie einige Beispiele hören. Anschließend werden Sie vor der Hauptaufgabe die Aufgabe üben. Bitte lesen Sie die Anweisungen sorgfältig durch. |
| The expected duration for DAUERDISKRIMINIERUNG task is ~ 30 minutes (7,5 minutes x 4 blocks with breaks in between) | Die voraussichtliche Dauer für die Aufgabe DAUERDISKRIMINIERUNG beträgt ~ 30 Minuten (7,5 Minuten x 4 Blöcke mit Pausen dazwischen) |
| You can travel between pages with the arrow keys freely until you learn the task. | Sie können mit den Pfeiltasten frei zwischen den Seiten wechseln, bis Sie die Aufgabe gelernt haben. |
| In each trial, you will first hear a rhythm consisting of 5 drum sounds, followed by a short pause. Then, you will hear another interval with 2 drum sounds. Your task is to decide whether this final interval is longer or shorter than the intervals making up the rhythm. If the final interval is LONGER, press L key. If it is SHORTER, press S key. | Bei jedem Versuch hören Sie zunächst einen Rhythmus, der aus 5 Trommelklängen besteht, gefolgt von einer kurzen Pause. Dann hören Sie ein weiteres Intervall mit 2 Trommelklängen.  Ihre Aufgabe ist es, zu entscheiden, ob dieses letzte Intervall länger oder kürzer ist als die Intervalle, aus denen der Rhythmus besteht.  Wenn das letzte Intervall LÄNGER ist, drücken Sie die Taste L. Wenn es KÜRZER ist, drücken Sie die Taste S. |
| Next page / Previous page | Nächste Seite / Vorherige Seite |
| Here is a graphical representation of one trial: You will first hear the drum sounds, then respond on the keyboard. The end of the trial will be signaled by a screen color change. | Hier sehen Sie eine grafische Darstellung eines Versuchs: Zuerst hören Sie die Trommelklänge, dann antworten Sie mit der Tastatur. Das Ende des Versuchs wird durch einen Farbwechsel des Bildschirms signalisiert. |
| Please do not count, and do not get help from any movement in your body while doing this task. | Zählen Sie bitte nicht und lassen Sie sich bei dieser Aufgabe nicht von einer Bewegung Ihres Körpers helfen. |
| If you understood the task,  please press ENTER to listen to the examples. | Wenn Sie die Aufgabe verstanden haben,  drücken Sie bitte die EINGABETASTE, um sich die Beispiele anzuhören. |
| You cannot go back to the current section once you proceed. | Sie können nicht zum aktuellen Abschnitt zurückkehren, wenn Sie fortfahren. |
| Press S key when the interval between the final pair of sounds is SHORTER than the ones making up the rhythm. | Drücken Sie die Taste S, wenn das Intervall zwischen dem letzten Tonpaar KÜRZER ist als das Intervall zwischen den Tönen, die den Rhythmus bilden. |
| Press L key when the interval between the final pair of sounds is LONGER than the ones making up the rhythm. | Drücken Sie die Taste L, wenn das Intervall zwischen dem letzten Tonpaar LÄNGER ist als das Intervall zwischen den Tönen, die den Rhythmus bilden. |
| You have completed the instructions.  In the following section, you will practice the task for at least 6 trials. At the end of each trial, you will be given feedback regarding your responses. | Sie haben die Anweisungen abgeschlossen.  Im folgenden Abschnitt werden Sie die Aufgabe für mindestens 6 Versuche üben. Am Ende eines jeden Versuchs erhalten Sie eine Rückmeldung zu Ihren Antworten. |
| When you are ready, please press ENTER to proceed | Wenn Sie bereit sind, drücken Sie bitte die EINGABETASTE, um fortzufahren. |
| Your response was correct. | Ihre Antwort war richtig. |
| The final interval was shorter than the previous intervals. Your response was incorrect. | Das letzte Intervall war kürzer als die vorherigen Intervalle. Ihre Antwort war falsch. |
| The final interval was longer than the previous intervals. Your response was incorrect. | Das letzte Intervall war länger als die vorherigen Intervalle. Ihre Antwort war falsch. |
| You were too late to respond.  Please try to respond fast and accurately. | Sie haben zu spät geantwortet.  Bitte versuchen Sie, schnell und genau zu antworten. |
| You have completed the practice section.  If you have questions, please notify the experimenter. | Sie haben den Übungsteil abgeschlossen.  Wenn Sie Fragen haben, informieren Sie bitte den Versuchsleiter. |
| Otherwise, please press ENTER to proceed to the main task | Andernfalls drücken Sie bitte die EINGABETASTE, um mit der Hauptaufgabe fortzufahren. |
| Note that no feedback will be given throughout this section | Beachten Sie, dass während dieses Abschnitts kein Feedback gegeben werden kann. |
| You have successfully completed the DURATION DISCRIMINATION section. | Sie haben den Abschnitt DURATION DISCRIMINATION erfolgreich abgeschlossen. |
| You can take a short break before the next section. | Sie können vor dem nächsten Abschnitt eine kurze Pause einlegen. |
| Press ENTER to end the break  and proceed to the next section. | Drücken Sie die EINGABETASTE, um die Pause zu beenden  und fahren Sie mit dem nächsten Abschnitt fort. |
| BREAK. | PAUSIEREN. >> PAUSE |
| Now, you will repeat the task you completed at the beginning of the  experiment, where you tapped without listening to any sound. | Jetzt wiederholen Sie die Aufgabe, die Sie zu Beginn des Experiments durchgeführt haben, bei dem Sie getippt haben, ohne ein Geräusch zu hören. |

| **PREFERRED TEMPO** | **BEVORZUGTES TEMPO** |
| --- | --- |
| SLIDER | SCHIEBEREGLER |
| In the following trials, you will hear a continuous rhythm that will start with either fast or slow tempo. | Bei den folgenden Versuchen; hören Sie einen fortlaufenden Rhythmus, der entweder mit schnellem oder langsamem Tempo beginnt. |
| Your task is to click on different locations on the slider to change the tempo, and finally, to indicate the tempo that you prefer the most (you are most comfortable with). | Ihre Aufgabe ist es, auf verschiedene Stellen des Schiebereglers zu klicken, um das Tempo zu ändern, und schließlich das Tempo anzugeben, das Ihnen am besten gefällt (mit dem Sie sich am wohlsten fühlen). |
| Press ENTER to proceed to the task. | Drücken Sie ENTER, um mit der Aufgabe fortzufahren. |
| Click on the left button  to CHANGE the tempo | Klicken Sie auf die linke Taste, zum Ändern des Tempos |
| Click on the left button  to SET the tempo to a comfortable rate | Klicken Sie auf die linke Taste  um das Tempo auf eine angenehme Geschwindigkeit einzustellen |

| KEYPRESS | TASTENDRUCK |
| --- | --- |
| In the following trials, you will hear a continuous rhythm that will start with either fast or slow tempo and change its tempo gradually. | In den folgenden Versuchen hören Sie einen fortlaufenden Rhythmus, der entweder mit einem schnellen oder einem langsamen Tempo beginnt und sein Tempo schrittweise ändert. |
| Your task is to press ENTER when the tempo is not too fast or too slow but is at a comfortable rate that feels ‘just right’. | Ihre Aufgabe ist es, die EINGABETASTE zu drücken, wenn das Tempo nicht zu schnell oder zu langsam ist, sondern eine angenehme Geschwindigkeit hat, die sich "genau richtig" anfühlt. |
| A circle will show how much time is left to give a response. Please specify your preferred tempo before the circle disappears. | Ein Kreis zeigt an, wie viel Zeit noch für eine Antwort bleibt. Bitte geben Sie Ihr bevorzugtes Tempo an, bevor der Kreis verschwindet. |
| The next trial will play automatically once you press enter. Please get ready and press ENTER to continue. | Der nächste Versuch wird automatisch abgespielt, sobald Sie die Eingabetaste drücken. Bitte machen Sie sich bereit und drücken Sie ENTER, um fortzufahren. |
| Press ENTER when the tempo is not too fast or too slow but is at a comfortable rate that feels ‘just right’. | Drücken Sie die EINGABETASTE, wenn das Tempo nicht zu schnell oder zu langsam ist, sondern ein angenehmes Tempo hat, das sich "genau richtig" anfühlt. |
| There was no response. Press ENTER to try again. | Es gab keine Antwort. Drücken Sie ENTER, um es erneut zu versuchen. |
| The next trial will play automatically once you press enter. Please get ready and press ENTER to continue. | Der nächste Versuch wird automatisch abgespielt, sobald Sie die Eingabetaste drücken. |
| You have completed the experiment.  Thank you for your participation. | Sie haben das Experiment abgeschlossen. Vielen Dank für Ihre Teilnahme. |
| Press ENTER to exit. | Drücken Sie die EINGABETASTE zum Beenden. |
